# Sex-Related Differences in Patients’ Characteristics, Provided Care, and Outcomes Following Spontaneous Intracerebral Hemorrhage

**DOI:** 10.1007/s12028-022-01453-y

**Published:** 2022-04-07

**Authors:** Sophie Shih-Yüng Wang, Stefan Yu Bögli, Nathalie Nierobisch, Stella Wildbolz, Emanuela Keller, Giovanna Brandi

**Affiliations:** 1 Department of Neurosurgery and Neurotechnology, Eberhard Karls University Tübingen, Hoppe-Seyler-Strasse 3, 72070 Tübingen, Germany; 2 Institute for Intensive Care Medicine, University Hospital Zurich, Zurich, Switzerland; 3 Department of Neurology, University Hospital Zurich, Zurich, Switzerland; 4 Department of Diagnostic and Interventional Radiology, University Hospital of Zurich, Zurich, Switzerland; 5 Department of Neurosurgery and Clinical Neuroscience Center, University Hospital and University of Zurich, Zurich, Switzerland

**Keywords:** Intracerebral hemorrhage, Gender medicine, Stroke, Intensive care

## Abstract

**Background:**

Sex-related differences in patients with spontaneous, non-traumatic intracerebral hemorrhage (ICH) are poorly investigated so far. This study elucidates whether sex-related differences in ICH care in a neurocritical care setting exist, particularly regarding provided care, while also taking patient characteristics, and outcomes into account.

**Methods:**

This retrospective single center study includes all consecutive patients with spontaneous ICH admitted to the neurocritical care unit in a 10-year period. Patients’ demographics, comorbidities, symptoms at presentation, radiological findings, surgical and medical provided care, intensive care unit mortality and 12 month-mortality, and functional outcome at discharge were compared among men and women.

**Results:**

Overall, 398 patients were included (male = 198 and female = 200). No differences in demographics, Charlson Comorbidity Index (CCI), symptoms at presentation, radiological findings, intensive care unit mortality and 12-month mortality were observed among men and women. Men received an external ventricular drain (EVD) for hydrocephalus-therapy significantly more often than women, despite similar location of the ICH and radiographic parameters. In the multivariate analysis, EVD insertion was independently associated with male sex (odds ratio 2.82, 95% confidence interval 1.61–4.95, *P* < 0.001) irrespective of demographic or radiological features. Functional outcome after ICH as assessed by the modified Rankin scale, was more favorable for women (*P* = 0.044).

**Conclusions:**

Sex-related differences in patients with ICH regarding to provided neurosurgical care exist. We provide evidence that insertion of EVD is associated with male sex, disregarding clear reasoning. A sex-bias as well as social factors may play a significant role in decision-making for the insertion of an EVD.

## Introduction

Sex-differences in patients with stroke has already been the subject of several studies: Spontaneous intracerebral hemorrhage (ICH) incidence and prevalence has been described to be higher in men than in women, whereas women are older in age then man when spontaneous intracerebral hemorrhage (ICH) occurs [1–5]. Higher prevalence of tobacco and alcohol abuse in men have been discussed as a possible reason for this kind of disparity [3, 6]. On the contrary, subarachnoid hemorrhages and cardioembolic strokes, seem to affect more women than men [7–9]. The comparison of functional outcome between both sexes remains controversial with conflicting data without a clear sex with superior functional outcome, especially if adjusted for age [3, 6, 10]. Parameters of provided care (i.e., interventions, surgeries, medication, etc.) give relevant hints to uncover sex differences in certain pathologies. These aspects of provided care have not yet been thoroughly investigated in spontaneous non traumatic ICH. A better understanding of sex-related differences in patients with ICH may allow clinicians to more accurately advise patients and next-of-kin on the risks and benefits following ICH as well as counterbalance sex-bias in surgical and intensive care treatment.

The present single-center study investigates, whether sex-related differences regarding provided care, patients’ characteristics, and clinical outcomes in this particular cohort of patients with primary non traumatic ICH admitted to a neurocritical care unit exist in a retrospective manner.

## Methods

This study was approved by the Ethical Committee in the Kanton of Zurich (IRB approval number KEK 2019-00.713). It was performed in accordance with the ethical standards as laid down in the 1964 Declaration of Helsinki and its later amendments. For this type of study, formal consent is not required. Strengthening of the Reporting of Observational Studies in Epidemiology (STROBE) guidelines were used to draft the article.

### Patients

All patients admitted to the neurocritical care unit of the University Hospital of Zurich between January 2009 and December 2019 with spontaneous ICH were eligible for inclusion in this retrospective study. Inclusion criteria of the study were (1) adults (aged > 18 years) and (2) presence of spontaneous ICH. Exclusion criteria were (1) patients’ written or documented oral refusal to have their data analyzed for research projects; (2) ICH secondary to cavernoma, dural arteriovenous fistula, other arteriovenous malformation, or histopathologically confirmed amyloidangiopathy (in autopsies and surgical biopsy during hematoma evacuation); (3) ICH of traumatic etiology; and (4) sole intraventricular hemorrhage with no parenchyma involvement. To rule out ICH of secondary genesis, computed tomography angiography is routinely performed in our department when the patient is admitted.

### Data Collection

Data were obtained from the hospitals’ electronic health records (KISIM-TM; Cistec,® Zurich, Switzerland). Demographic data collected were: sex, age, height, weight, and presence of comorbidities, based on the Charlson Comorbidity Index (CCI) (i.e., history of myocardial infarction, congestive heart failure, peripheral vascular disease, history of cerebrovascular event, dementia, chronic pulmonary disease, rheumatologic disease, gastric ulcer, liver disease, diabetes without or with chronic complications, kidney disease, and history of cancer) [11]. Collected symptoms at presentation included hypertension (defined by a systolic blood pressure greater than 160 mmHg), headache, nausea and vomiting, and seizure at onset. Data concerning surgically provided care included insertion of an external ventricular drain (EVD), and surgical hematoma evacuation. At our institution the indication for EVD insertion is made by the treating neurosurgeon on the basis of imaging and their clinical expertise. Additionally, hematoma evacuation is performed by osteoplastic craniotomy only. Endoscopic evacuation is not commonly used at our institution. Moreover, primary decompressive craniectomy is not part of our institution’s treatment protocol. The following intensive care unit (ICU) complications were included: pneumonia (defined as new pulmonary infiltrate by chest X-ray, increased inflammatory blood parameters, and/or positive bacterial culture of tracheal secretion), clinical or electroencephalographic seizure, and blood- and/or platelets transfusion. Radiological findings on the initial cranial computer tomography (CT) scan included the main location of ICH, the presence of a spot sign, perifocal edema, subarachnoid hemorrhage, signs of ventriculomegaly (acute abnormal expansion of the cerebral ventricles), and hemorrhage volume. The hemorrhage volume was calculated using the formula of (abc)/2 (a = maximum length in mm, b = perpendicular width to a in mm, and c = number of slices multiplied by slice thickness in mm) from the initial CT scan. A neuroradiologist (NN) blinded to the clinical presentation and outcome evaluated all imaging. As outcome, ICU-length of stay (in days), hospital-length of stay (in days), mortality (30 days and 1 year after initial ICH), and functional outcome, as evaluated with the modified Rankin scale (mRS) at discharge were collected. Additionally, medical records and family interviews were then searched for indicators for early withdrawal of therapy (within 72 hours) and premorbid functional status (“independent” or “special care”) before the occurence of ICH and noted if it applied. “Independent” refers to autonomy in activities of daily living, life participation, social relationships and occupational performance if the patient is at pre-retirement age. “Special care” refers to any limitations in daily activities that make autonomous living at home impossible and require admission to a nursery home.

### Statistical Analysis

Statistical analysis was performed using SPSS V.25. Descriptive statistics are reported as counts/percentages, mean ± standard deviation, or median and interquartile range, as appropriate. For the analysis of associations, patient characteristics were dichotomized depending on either sex or the insertion of an EVD, or the outcome (mRS score 0–3 vs. mRS 4–6). All continuous data were tested for normality using Shapiro–Wilk’s test. Categorical variables were compared using Pearson’s χ^2^ test or Fisher’s exact test. Continuous/ordinal variables compared using Student’s t-test or Mann–Whitney U-test for parametric and non-parametric data, respectively, as appropriate. Multivariate binomial logistic regression was performed to ascertain the independence of the associations found on the likelihood of insertion of EVD or on the likelihood of unfavorable outcome in the prior univariate analysis. Multivariate binomial logistic regression was performed to ascertain the independence of the associations found on the likelihood of insertion of EVD in the prior univariate analysis. Significance was defined as the probability of a two-sided type 1 error being < 5% (*P* < 0.05).

## Results

**Fig. 1:**
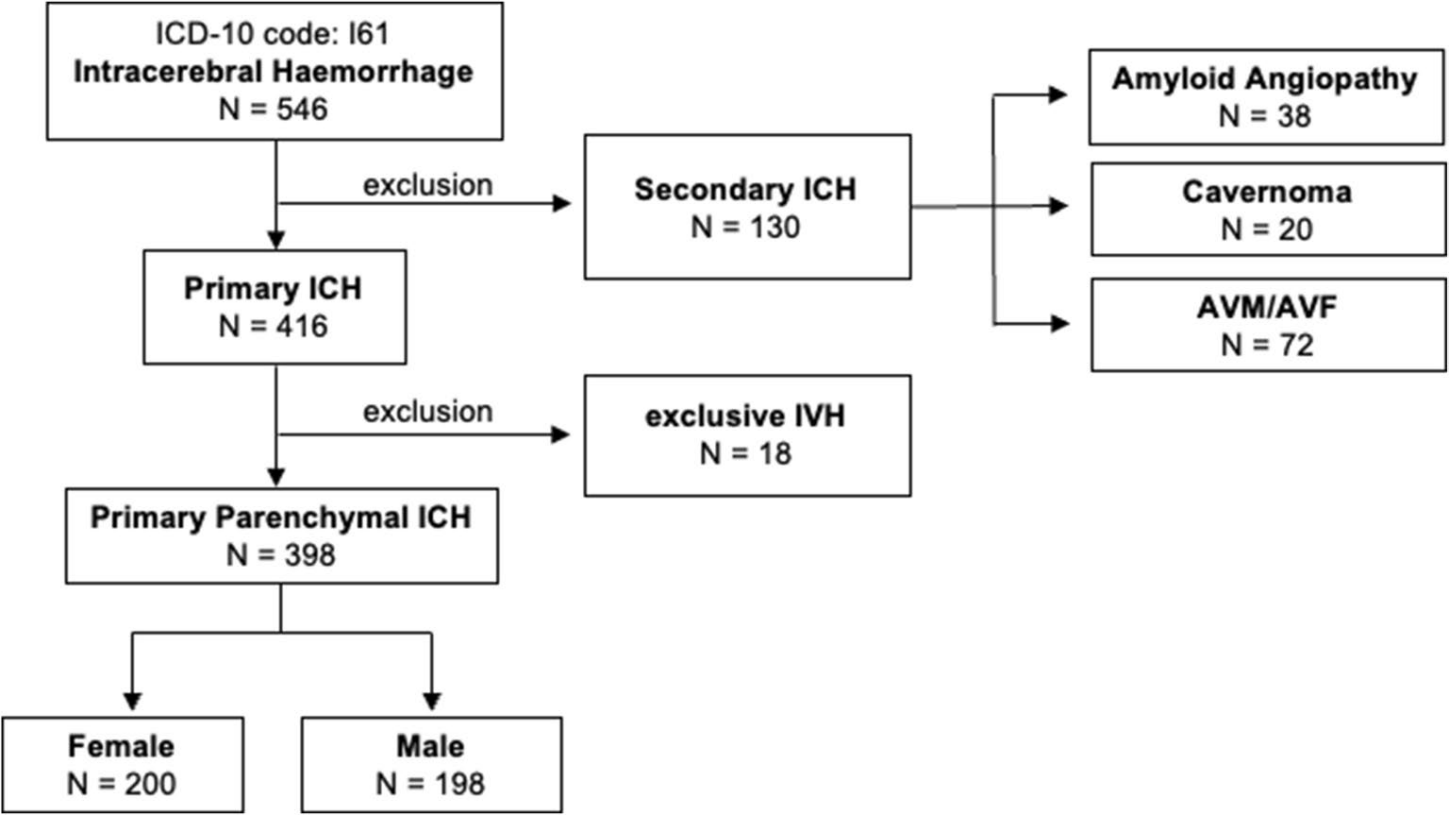
Flowchart of patients’ cohort

Overall, 548 patients were admitted to the neurocritical care unit with International Classification of Diseases, 10th Revision code I.61 for ICH during the study period. Of those, 398 patients fulfilled the inclusion criteria (Fig. 1). Thereof, 198 were male (49%) and 200 (51%) were female. Demographic characteristics, presence of comorbidities, symptoms at presentation, and scores at admission are available in Table 1. No differences in age and symptoms at presentation between both sexes were observed. CCI score did not differ among sexes, but women more often suffered from rheumatic disease (*P* = 0.049), whereas men more likely had diabetes (*P*  = 0.001) and prior myocardial infarction (*P* = 0.003).

**Table 1:** Demographic data, initial clinical features, comorbidities

	All (*N* = 398)	Female (*N* = 200)	Male (*N* = 198)	*P*-value
Age	66.14 ± 13.53	67.19 ± 13.52	65.09 ± 13.39	0.121
ICH score	1 (1, 2)	1 (1, 2)	1 (1, 2)	0.878
Initial GCS score	13 (8, 15)	13 (8, 15)	13 (8, 15)	0.782
Admission systolic blood pressure > 160 mmHg	139 (35%)	76 (38%)	63 (32%)	0.117
Initial headache	92 (23%)	44 (22%)	48 (24%)	0.504
Inital nausea and vomiting	106 (27%)	52 (26%)	54 (27%)	0.607
Seizure at onset	41 (10%)	18 (9%)	23 (12%)	0.312
History of tobacco use	70 (18%)	26 (13%)	44 (22%)	**0.042**
Anticoagulation use	91 (23%)	41 (20%)	50 (25%)	0.26
*Charlson Comorbidity index score*	1.78 ± 2.37	1.63 ± 1.86	1.93 ± 2.78	0.201
History of myocardial infarction	33 (9%)	8 (4%)	25 (13%)	**0.003**
Congestive heart failure	41 (10%)	21 (10%)	20 (10%)	1
Peripheral vascular disease	31 (8%)	11 (5%)	20 (10%)	0.127
History of cerebrovascular event	56 (14%)	27 (13%)	29 (15%)	0.918
Dementia	23 (6%)	12 (6%)	11 (6%)	1
Chronic pulmonary disease	36 (9%)	13 (6%)	23 (12%)	0.109
Rheumatologic disease	27 (7%)	19 (9%)	8 (4%)	**0.049**
Gastric ulcer	3 (1%)	1 (1%)	2 (1%)	0.993
Liver disease	12 (3%)	6 (3%)	6 (3%)	0.58
Diabetes (all)	69 (17%)	21 (10%)	48 (24%)	** < 0.001**
Mild-moderate diabetes	48 (12%)	13 (6%)	35 (18%)	**0.001**
Diabetes with chronic complications	5 (1%)	2 (1%)	5 (3%)	0.438
Kidney disease	78 (20%)	38 (19%)	40 (20%)	0.861
History of Cancer	40 (10%)	27 (13%)	13 (7%)	0.054

Bold values indicate statistical significance at *P* < 0.05

Values are indicated as number (percentage) mean ± standard deviation, or median interquartile range

*GCS* Glasgow Coma Scale, *ICH* intracerebral hemorrhage

### Radiological Findings

When we considered the hemorrhage site location, more women than men presented with lobar ICH (*P* = 0.007). Within lobar ICH, women were more likely to present with frontal and temporal ICH, and men presented more frequently with occipital hemorrhages. In addition, the frequency of deep ICH (basal ganglia or thalamus) did not differ among men and women (*P* = 0.224). Similarly, the frequency of the infratentorial ICH (e.g. brainstem or cerebellum) did not differ between sexes. Hemorrhage volume, hematoma expansion, presence of subarachnoid hemorrhage, ventriculomegaly, perifocal edema, intraventricular hemorrhage, and midline shift of parenchyma the ventricle on the first head CT-scan were not significantly different between groups (Table 2).

**Table 2:** Radiographic features

	All (*n* = 398)	Female (*n* = 200)	Male (*n* = 198)	*P*-value
Ventriculomegaly	165 (41)	88 (44)	75 (38)	0.299
Subarachnoid hemorrhage	211 (53)	111 (55)	100 (51)	0.369
Perifocal edema	369 (91)	186 (93)	177 (89)	0.365
Hemorrhage volume (mL)	40.32 ± 47.71	39.32 ± 46.54	41.33 ± 48.96	0.675
Hematoma expansion	151 (38)	87 (44)	64 (32)	0.243
Midline shift parenchyma (mm)	7.87 ± 4.76	8 ± 4.77	7.71 ± 4.76	0.664
Midline shift ventricle (mm)	7.42 ± 4.76	7.49 ± 4.99	7.34 ± 4.49	0.823
Ventricular extension	246 (62)	124 (62)	122 (62)	0.97
Intraventricular hemorrhage including- third and fourth ventricle	178 (45)	93 (47)	85 (43)	0.482
Progredient ventricles	140 (35)	82 (41)	58 (29)	0.275
Localisation				**0.007**
Deep	172 (43)	78 (45)	94 (55)	
Lobar	140 (35)	81 (58)	59 (42)	
Infratentorial	86 (22)	41 (48)	45 (52)	
Deep				0.224
Basal ganglia	112 (28)	48 (43)	64 (57)	
Thalamus	60 (15)	30 (50)	30 (50)	
Lobar				**0.002**
Frontal	43 (11)	30 (70)	13 (30)	
Parietal	40 (10)	19 (48)	21 (52)	
Temporal	39 (10)	25 (64)	14 (36)	
Occipital	18 (5)	7 (39)	11 (61)	
Infratentorial				0.66
Cerebellum	52 (13)	24 (46)	28 (54)	
Brainstem	34 (9)	17 (50)	17 (50)	

Bold values indicate statistical significance at *P* < 0.05

Values are indicated as number and (percentage) and mean ± standard deviation, unless otherwise indicated

### Provided Care

Even though, seizures at onset were not more prevalent in women, they received anti-epileptic drugs significantly more often than men (*P* = 0.007). Among the other conservative medical treatments (such as administration of blood and/or platelet conserves, frequency of intubation and days on mechanical ventilation), no differences were found among men and women (Table 3).

**Table 3:** Provided care and outcomes

	All (*n* = 398)	Female (*n* = 200)	Male (*n* = 198)	*P*-value
EVD	112 (28)	45 (23)	67 (34)	**0.016**
Ventriculostomy related infection	13 (3)	4 (2)	9 (5)	0.153
Hematoma evacuation	90 (23)	49 (24)	41 (21)	0.433
EC given	32 (8)	16 (8)	16 (8)	1
TC given	61 (15)	33 (16)	28 (14)	0.51
Epilepsy during hospital stay	21 (5%)	13 (6%)	8 (4%)	0.28
Antiepileptic treatment	40 (10)	33 (16)	13 (7)	**0.007**
Intubation	173 (43)	88 (44)	85 (42)	0.75
Days on mechanical ventilation	4.63 ± 4.70	4.92 ± 5.46	4.38 ± 3.95	0.452
Pneumonia	88 (22%)	50 (25%)	38 (19%)	0.222
Length of ICU stay (days)	8.83 ± 21.22	6.93 ± 8.58	10.76 ± 28.74	0.073
Length of hospital stay (days)	15.08 ± 14.03	15.10 ± 12.97	15.05 ± 15.06	0.972
30 days mortaility	115 (29)	60 (30)	55 (28)	0.705
1 year mortality	142 (36)	73 (36)	69 (35)	0.575

Bold values indicate statistical significance at *P* < 0.05

Values are indicated as number (percentage) and mean ± standard deviation, unless otherwise indicated

*EVD* external ventricular drainage, *EC* Erythrocyte concentrate, *ICU* intensive care unit, *TC* Thrombocyte concentrate

When we considered surgical care, an EVD was inserted more often in men than in women (*P*-value = 0.016), whereas no differences were found in frequency of hematoma evacuation among men and women (*P* = 0.433) (Table 3). On the basis of the results of the univariate analysis evaluating differences depending on EVD insertion (Table 4), a multivariate analysis was performed (Table 5). Male sex, presence of intraventricular hemorrhage, ventriculomegaly, and volume as well as evacuation of ICH were independently associated with EVD insertion. Male sex in particular increased the odds of EVD insertion by 2.82 (95% confidence interval 1.61–4.95, *P* < 0.001).

**Table 4:** Univariate analysis dichotomized by EVD insertion

	No EVD (*n* = 286)	EVD (*n* = 122)	*P*-value
Demographic data/clinical features			
Age	67.15 ± 13.91	63.58 ± 12.21	**0.018**
Sex (male)	131 (45.8)	67 (59.8)	**0.012**
ICH score	1 (1, 2)	2 (1, 2)	0.311
Initial GCS score	13.5 (8.75, 15)	12 (6, 14)	**0.008**
Admission systolic blood pressure > 160 mmHg	104 (36.4)	35 (31.3)	0.336
Initial headache	67 (23.4)	25 (22.3)	0.814
Inital nausea and vomiting	73 (25.5)	33 (29.7)	0.395
Initial seizure	29 (10.2)	13 (11.8)	0.635
Anticoagulation use	60 (21)	31 (28.6)	0.106
Charlson Comorbidity index score	1 [0, 3]	2 [0, 3]	0.510
Radiographic features			
Ventriculomegaly	91 (31.8)	71 (63.4)	** < 0.001**
Subarachnoid hemorrhage	149 (52.1)	62 (55.4)	0.558
Intraventricular hemorrhage	147 (51.4)	99 (88.4)	** < 0.001**
Intraventricular hemorrhage including- third fourth ventricle	91 (32)	87 (71)	** < 0.001**
Perifocal edema	260 (90.9)	102 (91.1)	0.960
Initial hemorrhage volume	44.23 ± 52.85	30.34 ± 28.81	**0.001**
Hematoma evacuation	49 (17.1)	41 (33.6)	** < 0.001**
Hematoma expansion	81 (32.5.)	35 (31.3)	0.810
Midline shift parenchyma	148 (51.7)	63 (56.3)	0.418
Ventricular progression	59 (23.6)	46 (41.1)	**0.001**
Localization			
Deep	96 (33.6)	72 (64.3)	** < 0.001**
Lobar	139 (45.1)	11 (9.8)	** < 0.001**
Infraten	61 (21.3)	29 (25.9)	0.328
Deep			
Basal Ganglia	76 (26.6)	52 (46.4)	** < 0.001**
Thalamus	46 (16.1)	41 (36.6)	** < 0.001**
Lobar			
Frontal	76 (26.6)	13 (11.6)	**0.001**
Parietal	62 (21.7)	4 (3.6)	**0.001**
Temporal	69 (24.1)	12 (10.7)	**0.003**
Occipital	29 (10.1)	3 (2.7)	**0.014**
Infratentorial			
Cerebellum	38 (13.3)	22 (19.6)	0.111
Brainstem	40 (14.0)	13 (10.7)	0.195

Bold values indicate statistical significance at *P* < 0.05

Values are indicated as number (percentage), mean ± standard deviation, or median and (interquartile range)

*ICH* intracerebral hemorrhage, *GCS* glasgow coma scale, *EVD* external ventricular drain

**Table 5:** Multivariate analysis

	OR	95% CI	*P*-value
Age (per unit increase)	0.98	0.96–1.00	0.058
Sex (male as reference)	**2.82**	**1.61–4.95**	** < 0.001**
Initial GCS score (per unit increase)	0.96	0.90–1.02	0.171
Ventriculomegaly (yes as reference)	**2.94**	**1.63–5.32**	** < 0.001**
Volume hemorrhage (per mL increase)	**0.98**	**0.97–0.99**	** < 0.001**
Hematoma evacuation (yes as reference)	**7.97**	**3.70–17.17**	** < 0.001**
Intraventricular hemorrhage (yes as reference)	**7.45**	**3.57–15.54**	** < 0.001**
Localization (deep/ lobar/ infratentorial)	1.25	0.83–1.87	0.288

Bold values indicate statistical significance at *P* < 0.05

*GCS* Glasgow Coma Scale

Data are presented as odds ratio (OR) with 95% confidence interval (95%CI)

### Outcomes and Survival

**Fig. 2:**
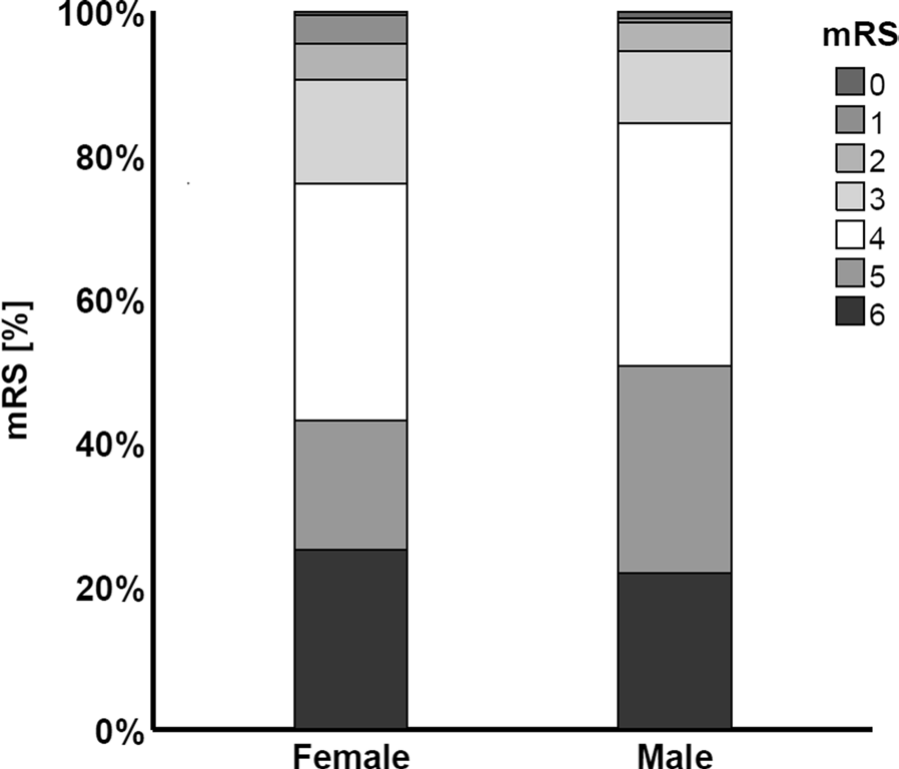
Modified Rankin scale at hospital discharge after the intracerebral hemorrhage

Length of ICU-stay, length of hospital stay, 30 day- and 1 year- mortality did not differ between groups (Table 3). When we compared the outcome as evaluated using the mRS, men were more likely to suffer from an unfavorable outcome (Fig. 2). Further predictors of an unfavorable outcome at hospital discharge are shown in Tables 6 and 7. The multivariable analysis (Table 8) showed higher age, male sex, lower initial GCS score, midline shift, insertion of EVD, and higher CCI to be predictive of unfavorable outcome independent of TC transfusion, presence of ventriculomegaly, presence of intraventricular hemorrhage volume of hemorrhage and localization of the hematoma.

**Table 6:** Demographic data, initial clinical features, comorbidities, provided care depending on outcome

	mRS 0–3 (*n* = 79)	mRS 4–6 (*n* = 319)	*P*-value
Age	63.00 ± 14.72	66.92 ± 13.13	**0.021**
Sex (Male)	31 (39)	167 (52)	**0.044**
ICH score	2 (1, 3)	2 (1, 3)	0.997
Initial GCS score	15 (14, 15)	12 (6, 14)	** < 0.001**
Admission systolic blood pressure > 160 mmHg	27 (34)	112 (35)	0.896
Initial Headache	17 (22)	75 (24)	0.767
Inital nausea and vomiting	17 (22)	89 (28)	0.260
Initial seizure	10 (13)	32 (10)	0.538
History of tobacco use	16 (20)	54 (17)	0.510
Anticoagulation use	17 (22)	75 (24)	0.767
Charlson Comorbidity Index score	0 (0, 2)	2 (0, 3)	**0.001**
EVD	9 (11)	103 (32)	** < 0.001**
Ventriculostomy-related infection	2 (3)	11 (3)	0.753
VP-shunt	0 (0.0)	18 (6)	0.030
Ventriculostomy-related infection	2 (3)	11 (3)	0.752
EC given	7 (9)	25 (8)	0.817
TC given	18 (23)	42 (13)	**0.035**
Antiepileptic treatment	7 (9)	39 (12)	0.440
Intubation	14 (18)	157 (49)	** < 0.001**
Pneumonia	17 (22)	71 (22)	0.882
Length of ICU stay	4.96 ± 5.97	9.79 ± 23.43	0.070
Length of hospital stay	13.22 ± 10.33	15.54 ± 14.79	0.188

Bold values indicate statistical significance at *P* < 0.05

Values are indicated as number (percentage), mean ± standard deviation, or median (interquartile range)

*EC* erythrocyte concentrate, *EVD* external ventricular drain, *GCS* Glasgow Coma Scale, *ICH* intracerebral hemorrhage, *ICU* intensive care unit, *mRS* modified Rankin scale, *TC* thrombocyte concentrate

**Table 7:** Radiographic Features depending on outcome

	mRS 0–3 (*n* = 79)	mRS 4–6 (*n* = 319)	*P* value
Ventriculomegaly	20 (25)	142 (45)	**0.002**
Subarachnoid hemorrhage	39 (49)	172 (54)	0.529
Intraventricular hemorrhage	38 (48)	208 (65)	**0.006**
Intraventricular hemorrhage including third fourth ventricle	25 (26)	153 (48)	**0.011**
Perifocal edema	65 (82)	297 (93)	**0.005**
Initial hemorrhage volume	20.30 ± 23.18	45.28 ± 50.85	** < 0.001**
Hematoma evacuation	22 (28)	68 (21)	0.230
Hematoma expansion	19 (24)	97 (30)	0.167
Midline shift parenchyma	25 (32)	186 (58)	** < 0.001**
Ventricular progression	13 (17)	92 (29)	**0.010**
Localisation			
Deep	10 (13)	158 (50)	** < 0.001**
Lobar	40 (51)	100 (31)	** < 0.002**
Infratentoriel	29 (37)	61 (19)	** < 0.001**
Deep			
Basal ganglia	6 (8)	122 (38)	** < 0.001**
Thalamus	2 (3)	85 (27)	** < 0.001**
Lobar			
Frontal	11 (14)	78 (25)	0.050
Parietal	12 (15)	54 (17)	0.740
Temporal	22 (28)	59 (19)	0.085
Occipital	10 (13)	22 (7)	0.106
Infratentoriel			
Cerebellum	22 (28)	38 (12)	**0.001**
Brainstem	9 (11)	43 (14)	0.769

Bold values indicate statistical significance at *P* < 0.05

Values are indicated as number (percentage) and mean ± standard deviation, unless otherwise indicated

*mRS* modified Rankin scale

**Table 8:** Multivariable analysis for unfavorable outcome

	*P*-value	OR	95% C.I
Age (per unit increase)	**0.002**	**1.04**	**1.01–1.06**
Sex (male as reference)	**0.012**	**2.19**	**1.19–4.03**
Initial GCS score (per unit increase)	** < 0.001**	**0.74**	**0.65–0.84**
CCI (per unit increase)	**0.019**	**1.23**	**1.03–1.45**
EVD (yes as reference)	**0.002**	**4.19**	**1.73–10.17**
TC given (yes as reference)	0.560	0.80	0.38–1.70
Ventriculomegaly (yes as reference)	0.458	0.76	0.37–1.57
Intraventricular hemorrhage (yes as reference)	0.686	1.14	0.60–2.17
Volume hemorrhage (per mL increase)	0.102	1.01	0.99–1.03
Midline shift parenchyma (yes as reference)	**0.010**	**2.56**	**1.25–5.26**
Localisation (deep/lobar/infratentoriel)	0.287	0.79	0.52–1.22

Bold values indicate statistical significance at *P* < 0.05

Data is presented as OR with 95%CI

*CCI* Charlson Comorbidity Index, *CI* confidence interval, *EVD* external ventricular drain, *GCS* Glasgow Coma Scale, *OR* odds ratio, *TC* thrombocyte concentrate

From all deceased patients *n* = 115, *n* = 55 (47,0%) died after early withdrawal of life-sustaining therapy. Of them *n* = 25 were men (45%) (*P* = 0.500 compared to female). Premorbid “special care” functional status was comparable in both sexes: *n* = 6 in women (20%) and *n* = 6 in men (24%) (*P* = 0.617).

## Discussion

In our cohort of 398 consecutive patients admitted at the ICU following spontaneous ICH, we found that sex-related differences in outcome and provided care existed. In a multivariate analysis, male sex was independently associated with a poorer outcome, and insertion of EVD was significantly associated with male sex disregarding a clear radiographic or clinical reason. To the best of our knowledge, this difference in provided care (EVD insertion) is firstly reported in our study. We suggest that a gender-bias as well as social factors might have played a significant role in decision-making for the insertion of an EVD.

Patients’ demographics and severity scores at presentation and premorbid functional status were similar among men and women. Similarly, absolute CCI scores did not differ among groups. However, as reported in other studies, women more likely suffered from rheumatic disorders, whereas men were more likely to have diabetes and a history of myocardial infarction [12–14]. Despite the similarities in demographics and presentation, men were almost three times as likely to receive an EVD in comparison with women. To understand the reasoning for this difference, we tested the hypothesis that men more frequently suffered from ICH in locations that were very difficult to be reached surgically (i.e., basal ganglia and the brain stem). In patients with such deep ICH, occlusive ventriculomegaly is very common, and the insertion of an EVD might represent the only surgical possibility to control the intracranial pressure. Interestingly, the frequency of deep as well as infratentorial ICH was similar among men and women. Additionally, radiographic signs for hydrocephalus (ventriculomegaly), intraventricular hemorrhage, and progression of ventricular size or ventricular shift–as well-known reasons for an EVD-insertion- were comparable among men and women. Therefore, we conducted a multivariate analysis with insertion of an EVD as outcome, and we found that male sex was independently associated with EVD insertion. Strikingly, even after correction for clinical and radiological features, male sex increased odds of EVD insertion by almost threefold. The same disparity did not apply to the incidence of surgical hematoma evacuation. Additionally, the frequency of limitation of life-sustaining therapy (early withdrawal within 72 hours) was comparable among men and women, and therefore it does not seem to be the reason for this observed difference in provided care in EVD placement.

We can only speculate if the reason for this discrepancy is a form of “gender-bias”. Previous studies, indeed, reported that not only patients’ sex but also physicians’ sex might play a role in the decision-making process in health care, showing a trend for less aggressive treatments for female patients [15–17]. Women, furthermore, commonly have been shown to have less social support than men (often because more women are widowed) while older men are more likely to have a recognized representative (i.e. spouses, partners or children) when a decisions for an invasive medical treatment is necessary [18]. With the available data, we are not able to assess whether these factors, or also additional socioeconomic factors might have played a role in the decision to insert an EVD. The finding that females received anti-epileptic drugs significantly more often than males, despite a similar frequency of clinical or electroencephalographic seizures, also reveals a disposition to provide more conservative treatments for females in comparison to their male counterparts.

Despite the mentioned sex –related differences in provided care in favor of provided hydrocephalus-treatment by EVD in male patients, male sex was associated with significantly poorer outcome. Incidence of ventriculostomy-related infection was the same in both sexes, ruling out that this relevant EVD-related morbidity is causing poorer functional outcome in men. Previous findings concerning sex-related differences in mortality after ICH are conflicting: studies have reported that younger women (< 65 years old) with ICH had a lower mortality than men but also that women suffered from higher mortality and morbidity after ICH [19] [20]. In secondary ICH related to vascular abnormality, female sex was an independent risk factor for poor outcomes [21].

Concerning mortality, we did not observe that women fared worse than men did, probably because our study population differed from those in previous studies, with women not being older than men, suggesting that a combination of both sex and age may contribute to differences in outcome after ICH [10, 20]. Concerning functional outcome at hospital discharge after the ICH, however, female patients achieved a favorable outcome (mRS score 0–3) more often than male patients did, despite less aggressive treatment. This finding suggests that women could have had a favorable functional outcome even more likely, if they had received equal intensity of care as men. Although it is not possible to prove this by our study design, if we considered the efficacy of EVD in treating hydrocephalus, even more women might have had a favorable outcome had they received an EVD. However, because of due to the invasive nature of the treatment the opposite cannot be ruled out and an EVD insertion might carry unknown, outcome-relevant morbidity in men.

### Strength and limitations of this Study

The strength of this study lies in the use of a database of consecutive patients with spontaneous ICH during a 10-year period with several parameters collected. However, due to the retrospective nature of our study, our study design bears limitations. Firstly, this is a single center study. Therefore, the generalizability of the results may be limited because we cannot exclude detection and referral biases. While most patients with severe clinical symptoms are admitted to the Neurocritical Care Unit in our institution, some may have also been admitted to the in-house intermediate care unit and thus, not been included in our study. Furthermore, while patients with histopathologically proven cerebral amyloid angiopathy were excluded, due to the small number of biopsies/autopsies we cannot exclude the possibility that some cases have still been included. Furthermore, MRIs were not regularly performed to screen for microbleeds. Moreover, the lack of data regarding the decision-making process of the neurosurgeon to insert an EVD only allows speculations about the reason of the sex-related discrepancy in frequency of EVD-insertion. Moreover, pre-admission status could only be recorded in a retrospective manner and not based on modified Rankin Scale. Due to the retrospective study design and unavailable data on the initial ICP or its course, the effect of EVD insertion on ICP management cannot be judged in this study design.

## Conclusion

Sex-related differences in provided care of patients with spontaneous ICH exist. Men were almost three times more likely to receive an EVD in comparison with women. Neither demographical characteristics (including age and clinical presentation) nor imaging features (including ICH location, volume, and existence of ventriculomegaly) explained this discrepancy. These findings support the previously reported disposition to more aggressive treatments for men in comparison with women.
